# Transient Gene Expression in Serum-Free Suspension-Growing Mammalian Cells for the Production of Foot-and-Mouth Disease Virus Empty Capsids

**DOI:** 10.1371/journal.pone.0072800

**Published:** 2013-08-20

**Authors:** Ana Clara Mignaqui, Vanesa Ruiz, Sylvie Perret, Gilles St-Laurent, Parminder Singh Chahal, Julia Transfiguracion, Ayelén Sammarruco, Victoria Gnazzo, Yves Durocher, Andrés Wigdorovitz

**Affiliations:** 1 Instituto de Virología, Centro de Investigación en Ciencias Veterinarias y Agronómicas, Instituto Nacional de Tecnología Agropecuaria (INTA), Hurlingham, Buenos Aires, Argentina; 2 Consejo Nacional de Investigaciones Científicas y Tecnológicas, Ciudad Autónoma de Buenos Aires, Buenos Aires, Argentina; 3 Biotechnology Research Institute, National Research Council of Canada, Montreal, Quebec, Canada; Saint Louis University, United States of America

## Abstract

Foot-and-mouth disease (FMD) is a highly contagious disease of cloven-hoofed animals. It produces severe economic losses in the livestock industry. Currently available vaccines are based on inactivated FMD virus (FMDV). The use of empty capsids as a subunit vaccine has been reported to be a promising candidate because it avoids the use of virus in the vaccine production and conserves the conformational epitopes of the virus. In this report, we explored transient gene expression (TGE) in serum-free suspension-growing mammalian cells for the production of FMDV recombinant empty capsids as a subunit vaccine. The recombinant proteins produced, assembled into empty capsids and induced protective immune response against viral challenge in mice. Furthermore, they were recognized by anti-FMDV bovine sera. By using this technology, we were able to achieve expression levels that are compatible with the development of a vaccine. Thus, TGE of mammalian cells is an easy to perform, scalable and cost-effective technology for the production of a recombinant subunit vaccine against FMDV.

## Introduction

Foot-and-mouth disease (FMD) is a highly contagious disease of cloven-hoofed animals such as cattle, pigs, sheep and deer. The disease is endemic in many parts of the developing world and continues to pose a serious threat to livestock industries. It is of economic importance because the presence of the disease in developing countries results in severe restrictions to international trade and an outbreak in FMD-free countries can cause billionaire losses [1]. Prevention and eradication of the disease in a country requires sustained effort at significant cost. Vaccination is still a major strategy in developing countries to control FMD [2].

Foot-and-mouth disease virus (FMDV), a member of the family *Picornaviridae*, genus *Aphthovirus*, is a non-enveloped single positive-sense stranded RNA virus and the etiological agent of the disease [3]. Seven serotypes of FMDV (A, O, C, SAT1, SAT2, SAT3 and ASIA1) and multiple subtypes within each serotype have been described. Viral infection or vaccination with one serotype does not confer protection against other serotypes [4]. Thus, an update of the antigenic composition of the vaccine is required when new field strains appear.

The vaccine currently being used consists on chemically inactivated virus. To produce this vaccine, suspension growing baby hamster kidney-21 (BHK-21) cells are infected with virus and binary ethyleneimine is subsequently used for the inactivation process [2,5]. There are a number of disadvantages with its use, including the need for high-biosafety production facilities, the risk of incomplete inactivation of the virus, the problem of discriminating between vaccinated and infected animals and the fact that some serotypes and subtypes have problems to grow in cell cultures [6,7]. In order to address these problems, development of new vaccines is needed. Recombinant empty capsids are a promising alternative subunit vaccine against FMD because they are as immunogenic as virions but lack the infectious nucleic acid [8,9]

FMDV has an icosahedral capsid that is formed by the assembly of the structural proteins VP1, VP2, VP3 and VP4. The capsid precursor P12A is processed by the protease 3C to produce the structural proteins VP0, VP3 and VP1. One copy of each protein spontaneously form the protomer (5S), subsequently five protomers form the pentamer (12S) and finally twelve pentamers assemble into the empty capsid (75S) [10]. Cleavage of VP0 into VP2 and VP4 occurs upon encapsidation of genomic RNA into the empty capsids. The assembly of higher-order structures has been shown to be dependent on N-terminal myristoylation of the capsid precursor [11]. Subviral particles produced during virion morphogenesis can be isolated from infected cells and characterized by different sedimentation coefficients in sucrose gradients.

Since natural FMDV empty capsids have been shown to be as immunogenic as virions, a number of approaches have reported the use of empty capsids as an alternative vaccine either as DNA vaccines, recombinant viral vectors or subunit vaccines expressed in the baculovirus system [12–16]. In all of them, the assembly of structural proteins into empty capsids seems to occur only when the structural proteins are expressed in an adequate concentration [17,18]. The production of recombinant FMDV empty capsids in mammalian cells has been poorly explored, probably due to difficulties in the isolation of high level expressing stable cell lines. The problem with the classical means of producing recombinant proteins in mammalian cells may be because protease 3C cleaves not only the capsid precursor P12A but also cellular proteins [19,20]. Indeed, attempts to generate a recombinant cell line that stably expresses the necessary proteins for empty capsid assembly failed repeatedly, since protease 3C impairs the isolation of high expressing clones [21]. Transient transfection of mammalian cells is a well known procedure commonly used at small scale. However, during the last decade, the use of suspension-growing mammalian cells, economic and efficient transfection reagents and optimized expression vectors has allowed transient gene expression (TGE) to become a simple, scalable and powerful technology to generate large amounts of recombinant proteins within a short time period [22,23]. Moreover, for some applications, TGE has become a competitive alternative to the development of stable cell lines. As the genetic selection step is avoided, the protease 3C negative effect on cell growth does not represent a shortcoming. The use of TGE has been reported for the production of a subunit vaccine against respiratory syncytial virus, expressing the viral fusion protein of the virus [24]. Moreover, it has been recently reported the use of TGE for the generation of HIV-1 Gag virus like particles [25]. In both cases, the development of stable cell lines is impaired because the recombinant viral proteins are cytotoxic for cells.

In the present work, we evaluated TGE in serum-free suspension-growing mammalian cells using polyethylenimine (PEI) as transfection reagent for the production of a recombinant subunit vaccine against FMD. Two strategies were explored: transfection with the P12A3C expression cassette and co-transfection with plasmids encoding the individual structural proteins (VP0, VP3 and VP1). Yields attractive for industry were achieved using the P12A3C expression cassette. Furthermore, the recombinant empty capsids elicited a protective immune response in mice. Thus, TGE represents a suitable strategy for the production of FMDV empty capsids in mammalian cells.

## Materials and Methods

### Ethics Statement

All the experiments involving animals were performed in accordance to protocols approved by the INTA’s Ethical Committee of Animal Welfare (Permit numbers: 19/2011 and 16/2011). All efforts were made to minimize suffering.

### Cells and viruses

The human embryonic kidney 293 cell line stably expressing a truncated Epstein–Barr virus Nuclear Antigen-1 (293-6E) was grown in suspension in a serum-free F17 medium (Invitrogen, Carsband, CA) as previously described [26]. Cells were grown in suspension with agitation at 120 rpm in a humidified incubator at 37°C with 5% CO_2_. BHK-21 cell monolayers were maintained in Dulbecco’s modified Eagle medium (DMEM), supplemented with 2% fetal bovine serum (FBS) in a humidified incubator at 37°C with 5% CO_2_.

FMDV serotype A/Arg/01 inactivated (iFMDV) with binary ethyleneimine and purified by sucrose gradient was used as a positive control for protein characterization assays and to formulate experimental vaccines. Infectious virus was obtained from vesicles of experimentally infected cattle with passages in BHK-21 cells and was used for viral challenge. All experiments involving infectious virus were performed in INTA biosafety level 3A facilities.

### Plasmids

The recombinant plasmids used in this study were based on the pTT5 vector [27].

pTT5-P12A3C and pTT5-P12A were constructed by subcloning the P12A3C and P12A fragments from the pCI-P12A3C and pCI-P12A by digesting the pTT5 vector and the fragments with *Nhe*I and *Not*I enzymes [28].

For the generation of pTT5-VP0, pTT5-VP3 and pTT5-VP1 vectors, the corresponding genes were amplified from pTT5-P12A with specific primers (VP0fw: AAAGCT AGC GCC ACC ATG GGG GCT GGA CAA TCC AGC CCAG, VP0rev: AAAGGA TCC TCA CTC TTT CGA GGG GAG CTC GCCG, VP3fw: AAAGCT AGC GCC ACC ATG GGG ATC TTC CCT GTC GCG TGC, VP3rev: AAAGGA TCC TCA CTG CGG TCG GGG GTC AAT GGG GAG GC, VP1fw: AAAGAA TTC GCC ACC ATG ACC ACC GCT ACT GGG GAA TCAG and VP1rev: AAAGGA TCC TCA CAA AAG CTG TTT TTC GGG TGC AATG). The PCR products were digested with specific endonucleases and cloned into the pTT5 vector, which was digested using the same enzymes. All genes include a Kozak sequence located immediately upstream the start codon.

Plasmids were amplified in *Escherichia coli* (DH5α) grown in Circle Grown medium (MP Biomedicals, Solon, OH), supplemented with 50 µg/ml ampicillin. Plasmids were purified using MAXI prep columns (Qiagen, Mississauga, ON). Absorbances at 260 nm (A_260_) and 280 nm (A_280_) were measured for plasmid quantification. Only plasmid preparations with A_260_/ A_280_ ratio between 1.75 and 2.00 were used.

### Transfection

Suspension-growing 293-6E cells were transiently transfected using linear 25 kDa polyethylenimine (PEI) (Polysciences, Warrington, PA). Two days before transfection, cells were diluted in fresh medium at 0.45-0.5 × 10^6^ cells/ml. Cells were transfected with viability greater than 95%, at densities that were between 1.5 and 2 × 10^6^ cells/ml, the day of transfection. Plasmid DNA and PEI were separately diluted in complete serum free F17 medium at 1 and 2.5 µg/ml of culture to be transfected, respectively. PEI was added dropwise to DNA. The mixture was vortexed for 4 s and incubated at room temperature for 3 min. The DNA: PEI mixture was then added to the cells. Small-scale transfections were performed in six-well plates. Large-scale transfections were performed in 2 L shake flasks. After transfection, cells were harvested, resuspended in lysis buffer (50 mM HEPES pH 7.4, 150 mM NaCl, 1% Thesit, 0.5% NaDeoxycholate) and analyzed for protein expression. Use of PEI for transfection may be covered by existing intellectual property rights, including US Patent 6,013,240, European Patent 0,770,140, and foreign equivalents for which further information may be obtained by contacting licensing@polyplus-transfection.com.

### Cell counts

A Cedex Analyzer (Roche, Laval, Qc) automated cell counter was used to determine cell density and viability. The assay is based on the trypan blue exclusion method.

### Recombinant protein analyses

Cell lysates were separated by sodium dodecyl sulfate-polyacrylamide gel electrophoresis (SDS-PAGE). Separated proteins were transferred onto a nitrocellulose membrane, blocked and then incubated with anti-FMDV guinea pig serum. After several washes, membranes were incubated with horseradish peroxidase-conjugated anti-guinea pig goat serum (KPL). The reaction was visualized with an enhanced chemiluminescence method. Quantification of recombinant proteins in cell lysates was carried out by enzyme-linked immunosorbent assay (ELISA). Microtiter plates (Immunolon II) were coated with anti-FMDV rabbit serum (1/3000) in carbonate-bicarbonate buffer, pH 9.6, at 4^°^C overnight. After washing with PBS 0.1% Tween-20 and blocking with 1% ovoalbumin in phosphate-buffered saline (PBS) 0.1% Tween-20, cell lysates were added and incubated on plates at 37^°^C for an hour. Known amounts of purified iFMDV were two-fold serially diluted and added to the wells for standard curve generation. Plates were then incubated with anti-FMDV guinea pig serum, followed by horseradish peroxidase-conjugated anti-guinea pig goat serum (KPL). O-phenylenediamine-H2O2 was used as substrate. The reaction was stopped with sulfuric acid 12%. Absorbance was recorded at 492 nm (A_492_) in a microplate reader (Thermo Scientifics MultiskanFC).

Structural protein characterization was carried out by ELISA using four different monoclonal antibodies (MAbs 1-5, 2-4, 3-3 and 3-2) directed to conformational epitopes of FMDV A/Arg/01 [29].

Lysate of 293-6E cells transfected with pTT5-P12A3C and mock transfected were loaded onto a 45-15% sucrose gradient and centrifuged at 16,500 rpm 16 h in a SW28 rotor at 4^°^C. Fractions were collected and tested by ELISA. Inactivated FMDV A/Arg/01 was run at the same conditions and used as positive control. Absorbance of the fractions corresponding to the virus was also measured at 260 nm.

### Immunization of mice and detection of humoral immunity

BALB/c nine-week-old male mice (School of Veterinary Sciences, La Plata, Argentina) were randomly divided into five groups, with seven mice in each group.

Group 1 received no treatment, group 2 received lysates of mock transfected cells, group 3 received lysates of pTT5-P12A3C transfected cells containing about 500 ng of recombinant protein, group 4 received lysates of pTT5-P12A transfected cells (the same volume of lysate as group 3) and group 5 received 500 ng of purified FMDVi. Vaccines were formulated with ISA206 (MONTANIDE) as adjuvant in a proportion adjuvant: antigen 60:40. Mice were intraperitoneally (IP) inoculated with 0.3 ml of each formulation and boosted 21 days post inoculation (dpi). Sera were collected at 14 and 28 dpi for humoral immunity analysis.

Anti-FMDV antibodies from immunized mice serum were measured by ELISA. Immunolon II plates were coated with anti-FMDV rabbit serum in carbonate-bicarbonate buffer, pH 9.6, at 4^°^C overnight. After washing and blocking, iFMDV was added. Serial dilutions of mice serum samples were incubated on plates for half an hour. Anti-FMDV antibodies were detected using horseradish peroxidase-conjugated anti-mouse goat serum. O-phenylenediamine-H2O2 was used as substrate. The reaction was stopped with sulfuric acid 12%.

### Viral challenge

Protection against FMDV was assessed as previously described [30]. Briefly, mice were IP inoculated with 10^2^ TCID infectious FMDV A/Arg/01. Animals were anesthetized and bled by retro orbital route 24 h later. Heparinized blood was spread on BHK-21 cell monolayers. After virus adsorption, monolayers were washed with PBS. Fresh DMEM with 2% FBS was added and the cells were kept at 37^°^C for 72 h in a 5% CO_2_ incubator. Animals were considered protected if the cell monolayer did not present cytopathic effect after a blind passage. Percentage of protection was calculated as (protected mice/challenged mice) × 100.

### Recognition of recombinant proteins by bovine sera

Microtiter plates (Immunolon II) were coated with anti-FMDV rabbit serum (1/3000) in carbonate-bicarbonate buffer, pH 9.6, at 4^°^C overnight. After washing and blocking, known amounts of purified iFMDV and the same amount of recombinant proteins or mock transfected cell lysates were added and incubated on plates at 37^°^C for an hour. Plates were then incubated with sera from bovines vaccinated with the conventional FMD vaccine, followed by horseradish peroxidase-conjugated anti-bovine goat serum (KPL). O-phenylenediamine-H2O2 was used as substrate. The reaction was stopped with sulfuric acid 12%. Percentage of positivity was calculated as (A_492_ from recombinant protein or mock lysate/A_492_ from iFMDV) × 100.

## Results

**Figure 1:**
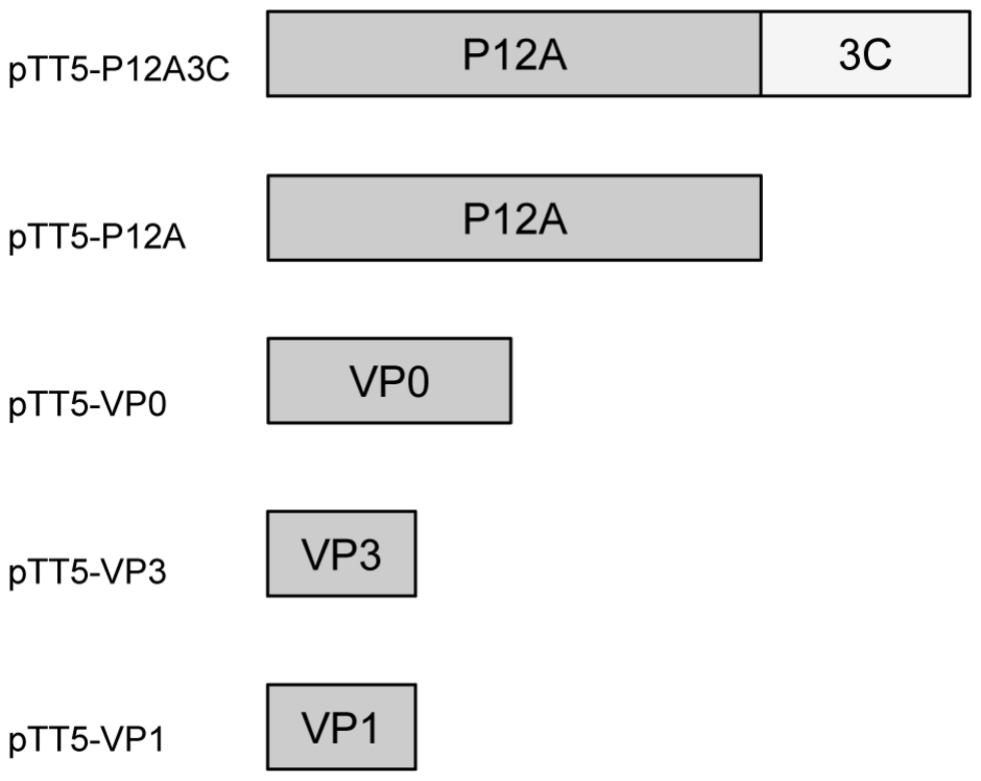
Schematic representation of FMDV genomic fragments cloned into the pTT5-vector.

The capsid precursor P12A and the protease 3C were cloned together (pTT5-P12A3C) and the structural proteins were cloned separately (pTT5-VP0, pTT5-VP3 and pTT5-VP1) (Figure 1).

Suspension growing 293-6E cells were transfected with pTT5-P12A3C or with the combination of pTT5-VP0, pTT5-VP3 and pTT5-VP1. Forty-eight hours post transfection (hpt) cells were harvested, resuspended in lysis buffer and analyzed for recombinant protein expression.

**Figure 2:**
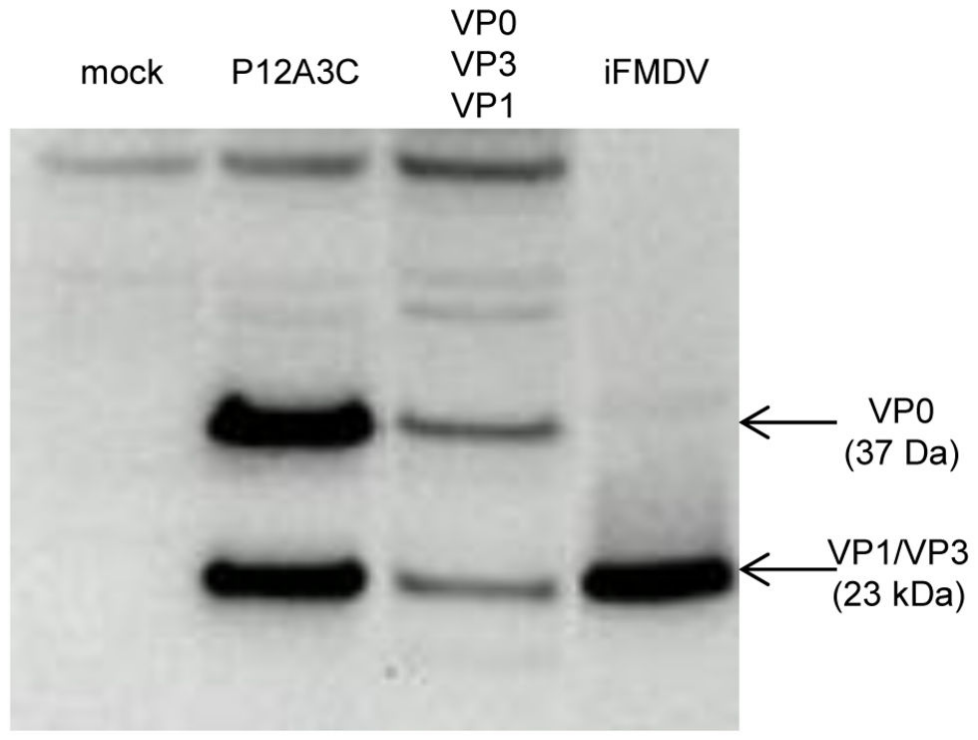
Western blot analysis of recombinant structural proteins. Lysates of cells transfected with pTT5-P12A3C, co-transfected with pTT5-VP0, pTT5-VP3 and pTT5-VP1 or mock transfected were analyzed by SDS-PAGE and Western blot using anti-FMDV guinea pig serum (1:500). iFMDV was used as positive control. The expected migration positions of VP0, VP3 and VP1 are indicated by arrows.

Western blot assay of the cell lysates showed that when the pTT5-P12A3C plasmid was used for transfection, the P12A capsid precursor was successfully cleaved by protease 3C into the structural proteins VP0 (37 kDa), VP3 (23 kDa) and VP1 (23 kDa) (Figure 2). The same band pattern was detected in the lysates of cells transfected with the plasmids encoding the individual structural proteins, confirming that the structural proteins were also successfully expressed.

The highest recombinant protein yield was obtained in the lysates of 293-6E cells transfected with pTT5-P12A3C. By using this strategy, the recombinant protein yield was 3 mg per liter of cell culture, measured by ELISA. Despite the absence of protease 3C, when cells were co-transfected with pTT5-VP0, pTT5-VP3 and pTT5-VP1, expression levels were 10 times lower than the ones obtained with pTT5-P12A3C.

**Figure 3:**
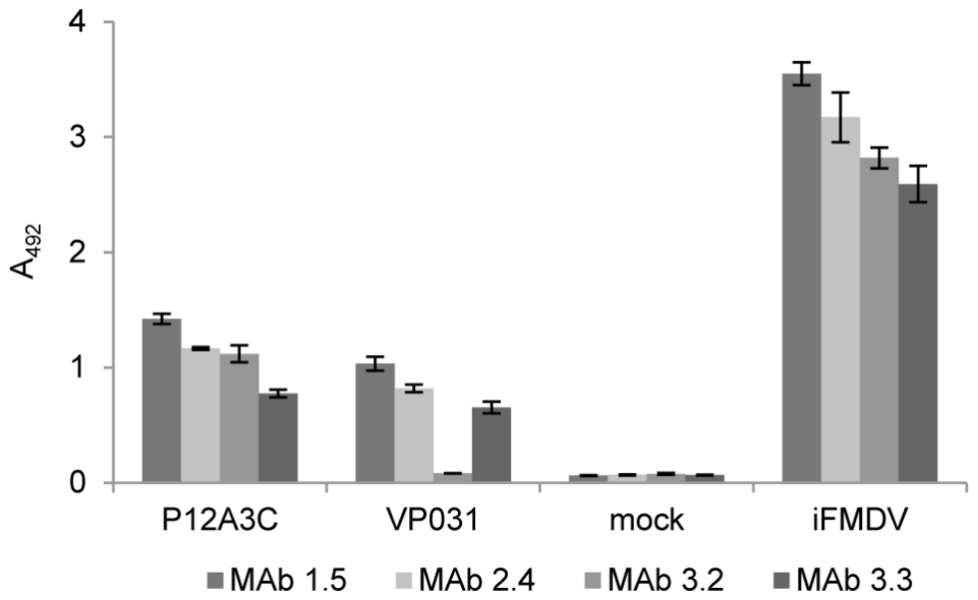
Antigenic reactivity of recombinant structural proteins. Lysates of cells transfected with pTT5-P12A3C, co-transfected with pTT5-VP0, pTT5-VP3 and pTT5-VP1, mock transfected cells or purified iFMDV were analyzed by ELISA with four MAbs (1–5,2–4,3–2,3–3) directed against conformational epitopes of FMDV. Error bars are standard deviation from three independent experiments.

In order to determine if the expressed proteins assembled into subviral particles, cell lysates were analyzed by ELISA using four monoclonal antibodies (MAbs) directed against conformational epitopes of the virus [29]. The four MAbs assayed reacted with cell lysates when the plasmid pTT5-P12A3C was used for transfection (Figure 3). Only three MAbs reacted with cell lysates when the plasmids encoding the individual structural proteins were used for transfection.

**Figure 4:**
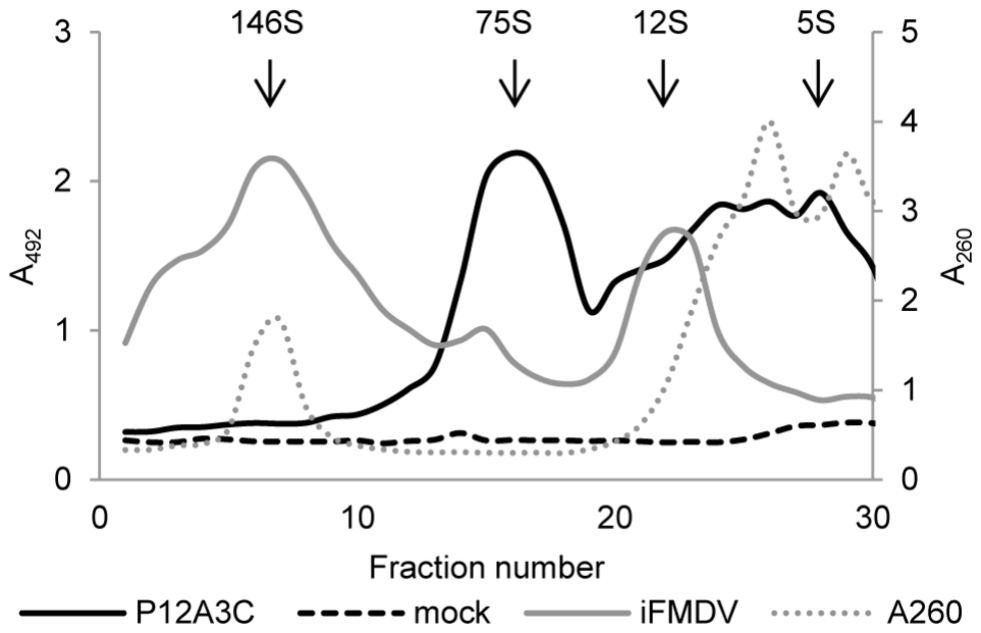
Assembly of recombinant structural proteins into subviral particles. Lysates of cells transfected with pTT5-P12A3C, mock transfected cells or iFMDV were loaded onto 45-15% sucrose gradients. Fractions were collected and analyzed by solid phase ELISA. Fractions of iFMDV were measured at 260 nm for RNA detection. The known positions of the FMDV virions (146S), empty capsids (75S), pentamers (12S) and protomers (5S) are indicated.

To further characterize the recombinant proteins, lysates of 293-6E cells transfected with pTT5-P12A3C were loaded onto 45-15% sucrose gradients. Inactivated FMDV A/Arg/01 was run in the same conditions as positive control. Lysates of cells transfected with pTT5-P12A3C showed a main peak of antigenic material that sedimented at the same rate as native empty capsids (75S). Antigenic peaks in the fractions corresponding to pentamers (12S) and protomers (5S) were also identified (Figure 4).

**Figure 5:**
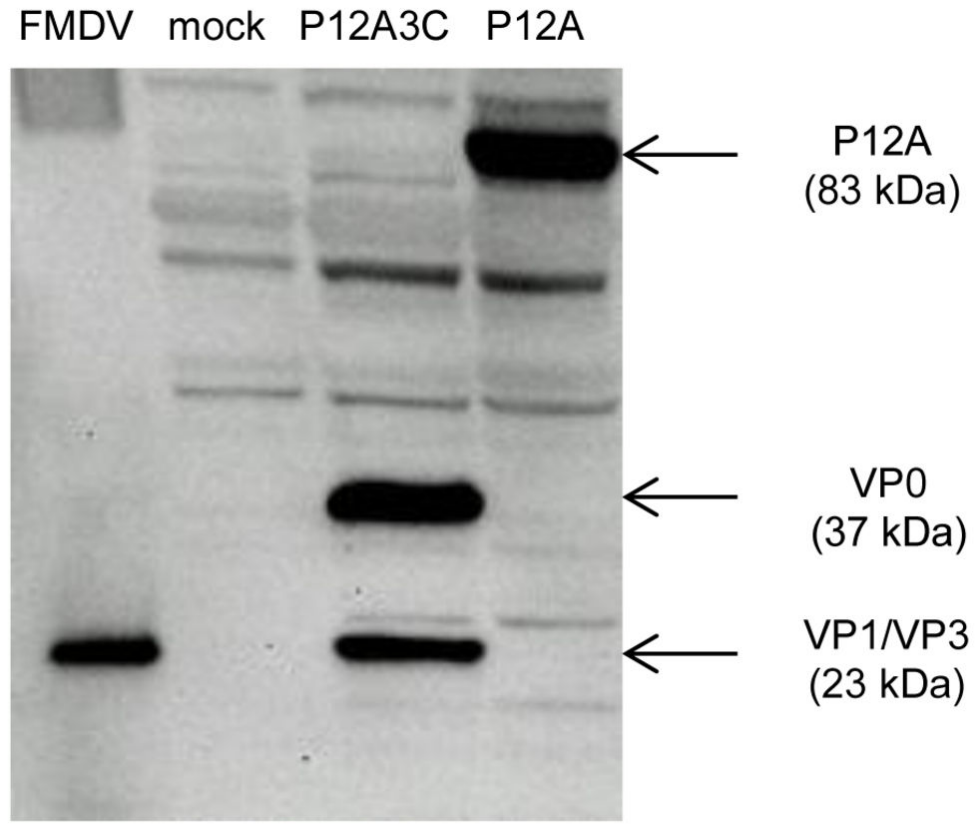
Western blot analysis of recombinant proteins produced after transfection with pTT5-P12A3C or with pTT5-P12A. Lysates of cells transfected with pTT5-P12A3C, pTT5-P12A or mock transfected were analyzed by SDS-PAGE and Western blot using anti-FMDV guinea pig serum (1:500). iFMDV was used as positive control. The expected migration positions of P12A, VP0, VP3 and VP1 are indicated by arrows.

**Figure 6:**
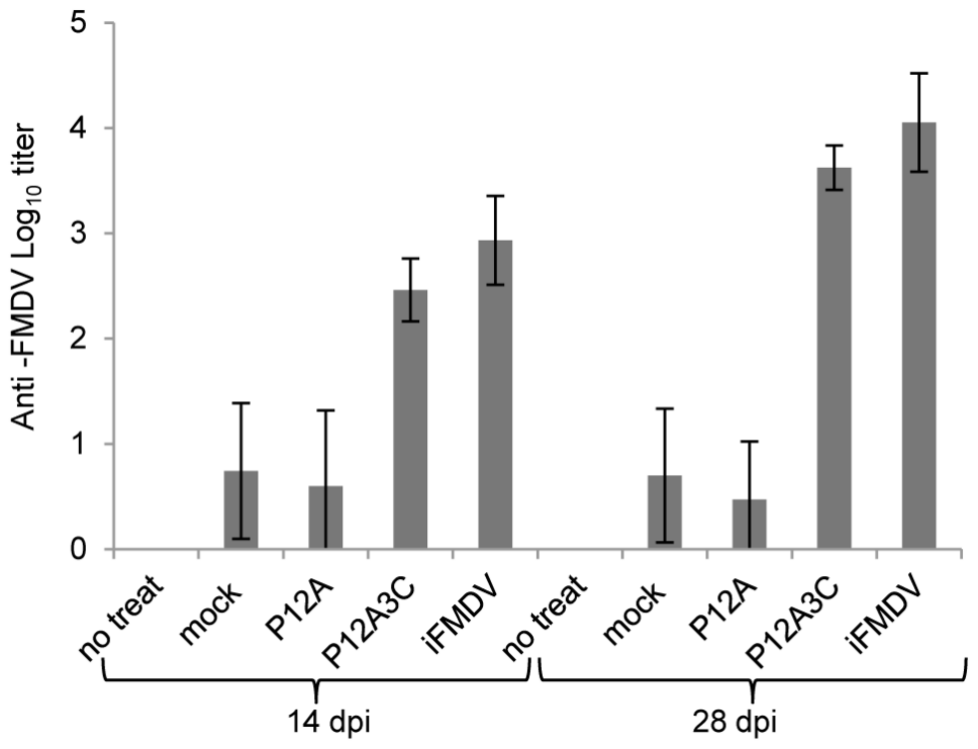
Analysis of humoral response of BALB/c mice immunized with recombinant proteins. The results were obtained from the average of seven sera in each group. Mean ± SD sample is shown.

For animal studies, 200 ml of cell culture were transfected with pTT5-P12A3C or with pTT5-P12A and harvested 48 hpt. Western blot assay showed that the P12A precursor was successfully expressed (83 kDa) and that a similar recombinant protein yield compared to the use of pTT5-P12A3C was achieved, based on band intensity (Figure 5). The capsid precursor P12A was expressed in order to elucidate whether the correct processing of the capsid precursor P12A and further assembly into subviral particles was a critical feature to elicit a protective immune response in mice. A significant difference was observed in the immune response elicited in mice vaccinated with proteins expressed from pTT5-P12A3C or pTT5-P12A, confirming that the correct cleavage of P12A by protease 3C and assembly of the structural proteins is critical to achieve a protective immunity. Mice inoculated with the lysates of cells transfected with pTT5-P12A3C and with inactivated FMDV induced high antibody serum titers at 14 dpi that increased after booster immunization (Figure 6). All these mice were protected against viral challenge (Table 1). Mice inoculated with the lysates of 293-6E cells transfected with pTT5-P12A and lysates of mock transfected cells, showed low serum titers that did not increase after booster immunization. However, a 43% of protection was obtained in mice inoculated with the recombinant capsid precursor P12A. Mice inoculated with the mock transfected cell lysates and those that did not received treatment, showed viraemia.

**Table 1:** Protection against viral challenge in BALB/c mice.

	No treatment	Mock transfected cells lysates	pTT5-P12A transfected cells lysates	pTT5-P12A3C transfected cells lysates	iFMDV
Protected/challenged^a^	0/6	0/7	3/7	6/6	7/7
% of protection^b^	0	0	43	100	100

^a^ Number of protected mice over number of challenged mice. Protection was established as absence of viremia 24 h post challenge.

^b^ The percentage of protection is calculated as (n^°^ of animals without viremia/n^°^ of challenged animals) × 100.

**Figure 7:**
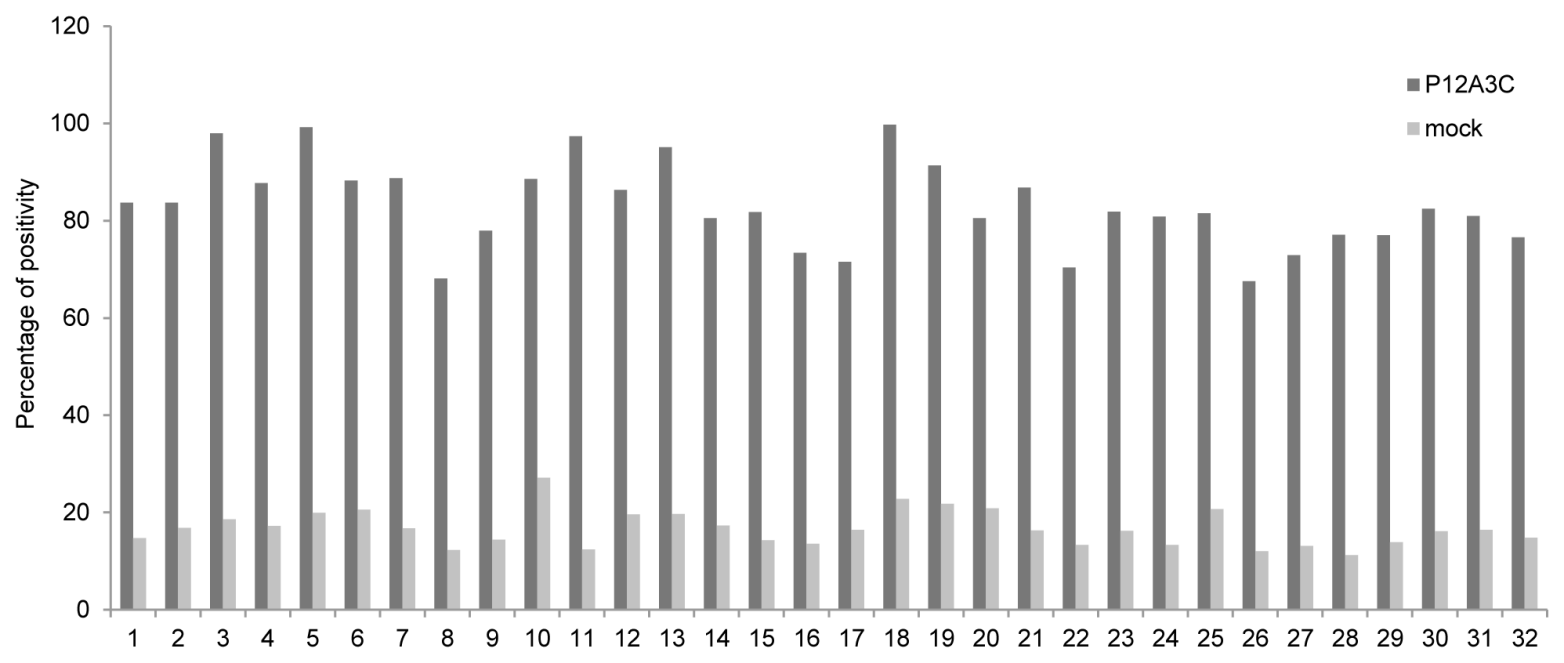
Recombinant empty capsids recognition by sera from vaccinated bovines. Percentage of positivity of bovine sera analyzed by ELISA for the recognition of recombinant empty capsids and mock transfected cell lysates. Percentage of positivity was calculated as (A_492_ from recombinant protein or mock lysate/A_492_ from iFMDV) × 100. Each pair of bars represents the sera from an individual animal.

Finally we proceeded to evaluate whether recombinant empty capsids were recognized by sera from cattle vaccinated with a commercial vaccine against FMDV. Sera from vaccinated bovines were assayed for the recognition of iFMDV, recombinant empty capsids and mock transfected cell lysates in a parallel ELISA assay. Sera from vaccinated bovines reacted similarly against recombinant empty capsids and iFMDV, and did not react with mock cell lysates (Figure 7). Sera from non-vaccinated bovines were also assayed, showing no reactivity with either the virus or recombinant empty capsids (data not shown).

## Discussion

When designing a new generation vaccine against FMDV, success not only depends on the immunogenicity of the novel vaccine, but also on the feasibility of the production strategy to be adopted by industrial laboratories. This feasibility depends on simplicity, scalability and versatility of the novel strategy proposed. This study was undertaken to evaluate TGE in serum-free suspension-growing 293-6E cells for the production of a recombinant subunit vaccine against FMD. For this purpose, plasmids encoding FMDV capsid precursor (P12A) together with protease 3C or encoding structural proteins individually (VP0, VP3 and VP1) were generated and transfected into 293-6E cells. The highest expression levels were achieved when pTT5-P12A3C plasmid was used for transfection. These levels are similar to the ones obtained in the vaccine facilities after infection of BHK-21 cells, representing an attractive yield to industrial laboratories [31]. P12A3C was also transiently expressed in BHK-21 cell monolayers using Lipofectamine 2000 (Invitrogen), but the yield was about 100 times lower when using pCI-neo vector [28]. Thus, the use of suspension-growing 293-6E cells in combination with the pTT5 vector is the key feature in this proposed method for the production of high amounts of FMDV recombinant proteins. This may be due to high cellular densities achieved in cell culture plus the episomal maintenance of the pTT5 plasmid in the transfected cells. Moreover, the use of serum-free medium and PEI as transfection reagent turns the proposed strategy into a cost-effective technology.

Even though protease 3C has a deleterious effect on protein expression [20,32], the strategy of co-transfecting cells with plasmids encoding the individual structural proteins (VP0, VP3 and VP1), resulted in lower expression levels compared to the use of the P12A3C expression cassette. This result is in accordance with Brautigam et al. [33] that reported the same result for poliovirus using the baculovirus expression system and could be related to mRNA instability. However, further studies should be done in order to confirm this hypothesis. The recombinant proteins expressed in 293-6E cells after co-transfection with pTT5-VP0, pTT5-VP3 and pTT5-VP1 assembled into structures that were not recognized by one of the MAbs directed against conformational epitopes of the virus. Thus, at least, one conformational epitope is absent. This difference in the assembly could be attributed to a tempo-spatial factor. Probably when the P12A3C expression cassette is used for transfection, after the proteolitic cleavage of the capsid precursor, the structural proteins are at the same time in a balanced concentration and close enough to interact properly assembling in the correct way. This issue remains to be further analyzed and could contribute to elucidate some steps of the assembly of picornavirus structural proteins that are incompletely understood. Thus, both the levels of expression and the quality of the recombinant protein are improved when cells are transfected with the P12A3C expression cassette.

It has been recently reported that optimal production of VP0, VP3 and VP1 in BHK-21 cells is achieved with reduced levels of protease 3C expression relative to P12A, compared to the use of P12A3C expression cassette [34]. However, when we co-transfected cells with 5% (w/w) of pTT5-3C and 90% of pTT5-P12A we achieved lower expression levels compared to the use of 90% of pTT5-P12A3C (data not shown).

Recombinant proteins expressed in 293-6E cells after transfection with pTT5-P12A3C assembled into empty capsids (75S) as well as pentamers (12S) and protomer (5S). These structures triggered an immune response, similar to the one elicited by the same amount of inactivated virus, that surpassed the viral challenge in a mice model. When recombinant capsid precursor P12A was used to immunize mice, no specific antibody response was detected. Since equimolar amounts of recombinant proteins were used for immunization, the only difference between both groups is the cleavage of the capsid precursor by protease 3C and further assembly into subviral particles of the structural proteins. This result, in agreement with previous studies, highlights the importance of the use of empty capsids when designing a FMD recombinant vaccine.

Despite the fact that mice inoculated with the recombinant capsid precursor P12A showed low serum titer levels that did not increase after booster immunization, 43% of protection was achieved. This protection could be due to cellular immunity, as already suggested [35] and should be further investigated.

Recombinant viral structures were recognized by sera from vaccinated bovines, further suggesting that recombinant structures mimic the authentic virus.

Our studies strongly suggest that TGE is a very promising approach for the production of a subunit vaccine against FMDV. Several alternative strategies to conventional vaccines, such as subunit vaccines, recombinant viral vectors and DNA vaccines, have been reported [36]. Most of these strategies are based on the use of the P12A3C expression cassette, either alone [13,37] or co-expressing different cytokines [16,38] or with modifications in the sequence in order to confer more stability to the capsid structure or less toxicity of protease 3C on the cells [12]. The production of recombinant empty capsids by TGE is the most similar strategy to the production process and the final formulation of the currently used inactivated vaccine. Instead of infecting suspension-growing BHK-21 cells, 293-6E cells should be transfected with PEI-DNA complexes eliminating the need of high biosafety containment facilities. The proposed technology is robust, inexpensive, easy to perform and scalable. Moreover, mammalian cells are the dominant system for the production of vaccines and therapeutic proteins. Thus, they represent a well known system for the industrial laboratories. The largest volume for TGE reported so far has been 100L [39]. Therefore, even though it would be difficult to replace the current inactivated vaccine in endemic countries because many doses are needed, FMD-free countries could produce emergency doses by TGE. This vaccine offers the advantage of safe production and fulfills the need to be able to demonstrate the re-establishment of the FMD-free status for trade purpose because final formulations are free from non-structural proteins and are compatible with so called DIVA tests that Differentiate Infection in Vaccinated Animals. Thus, with this technology FMD-free countries could adopt “vaccination to live” policies in case an outbreak occurs. The absence of serum in the cell culture diminishes the cost of the whole process and reduces the risk of adventitious virus to be present in the vaccine. Since only a cloning step is needed before production starts, TGE could allow the rapid incorporation of new field strains in the vaccine formulation and the production of those serotypes and subtypes difficult to adapt to cell culture. Furthermore, rationally designed modifications in the encoding sequences of the capsid precursor and protease 3C to stabilize the capsid structure or diminish the toxic effect of the protease, could be easily evaluated by TGE.

Traditionally, TGE evolved as an alternative approach to the generation of stable cell lines for the rapid production of mg of proteins for academia and preclinical studies. However, for toxic proteins -when the development of stable high expressing clones is not possible [21]- TGE seems to be the only rational and possible approach [24,25]. Moreover, with these proteins TGE can become a production system in its own right.

No therapeutic proteins produced by TGE have gain approval until now. In contrast with human vaccines, veterinary vaccines can be evaluated in target species making it easier to achieve the regulatory requirements needed for approval. Therefore, TGE represents a powerful and versatile technology for the production of recombinant FMDV empty capsids for vaccine development.

Future experiments should evaluate improvements in expression levels (for example by codon optimization or down regulation of protease 3C) and also explore the versatility of the technology for the expression of empty capsids of other serotypes of the virus. Also, additional studies are needed to investigate the immunogenicity of recombinant subviral particles in cattle.

## Conclusion

In conclusion, the use of TGE for the production of recombinant FMDV empty capsids represents a promising strategy for the development of a new generation vaccine.
